# Oral treatment with *Euterpe oleracea* Mart. (açaí) extract improves cardiac dysfunction and exercise intolerance in rats subjected to myocardial infarction

**DOI:** 10.1186/1472-6882-14-227

**Published:** 2014-07-08

**Authors:** Gisele Zapata-Sudo, Jaqueline S da Silva, Sharlene L Pereira, Pergentino JC Souza, Roberto S de Moura, Roberto Takashi Sudo

**Affiliations:** 1 Programa de Desenvolvimento de Fármacos, Instituto de Ciências Biomédicas, Universidade Federal do Rio de Janeiro, Rio de Janeiro, RJ, Brazil; 2 Departamento de Farmacologia e Psicobiologia, Instituto de Biologia Roberto Alcântara Gomes, Universidade do Estado do Rio de Janeiro, Rio de Janeiro, RJ, Brazil

**Keywords:** *Euterpe oleracea*, Myocardial infarction, Exercise intolerance, Left ventricular dysfunction, Cardiac hypertrophy

## Abstract

**Background:**

This study was designed to evaluate the cardioprotective effects of *Euterpe oleracea* Mart., popularly known as “açaí”, on rats subjected to myocardial infarction (MI).

**Methods:**

Hydroalcoholic extracts of açaí were obtained from a decoction of the seeds. Two male Wistar rat groups were delineated: 1) the sham-operated group (control, n = 6), with no surgical amendment, and 2) the MI group (n = 12), in which the anterior descendent coronary artery was occluded during surgery. MI group was divided into two subgroups, in which rats were either treated with hydroalcoholic extract of *Euterpe oleracea* seeds (100 mg/kg/day p.o.) or received no treatment. Treatment began on the day of surgery, and lasted 4 weeks. Subsequently, rats were subject to an exercise test protocol, hemodynamic evaluation, and histological analysis of the left ventricle. Groups were compared using one-way analysis of variance (ANOVA), followed by Dunnett’s test.

**Results:**

The total running distance of sham rats was 1339.0 ± 276.6 m, MI rats was 177.6 ± 15.8 m (*P* < 0.05), and MI-açaí rats was 969.9 ± 362.2 m. Systolic arterial pressure was significantly decreased in MI rats (86.88 ± 4.62 mmHg) compared to sham rats (115.30 ± 7.24 mmHg; *P* < 0.05). Açaí treatment prevented a reduction in systolic arterial pressure (130.00 ± 8.16 mmHg) compared to MI rats (*P* < 0.05). Left ventricular (LV) end-diastolic pressure was significantly augmented in MI rats (17.62 ± 1.21 mmHg) compared to sham rats (4.15 ± 1.60 mmHg; *P* < 0.05), but was 3.69 ± 2.69 mmHg in açaí-treated rats (*P* < 0.05 *vs.* MI). The LV relaxation rate (-dp/dt) was reduced in MI rats compared to the sham group, whereas açaí treatment prevented this reduction. Açaí treatment prevented cardiac hypertrophy and LV fibrosis in MI rats.

**Conclusions:**

*Euterpe oleracea* treatment of MI rats prevented the development of exercise intolerance, cardiac hypertrophy, fibrosis, and dysfunction.

## Background

Myocardial infarction (MI) is a major public health problem and the leading cause of human mortality in both developed and developing countries. MI is an acute condition of myocardial necrosis, which is caused by an imbalance between the coronary blood supply and myocardial demand. This condition is regularly followed by several biochemical alterations to the body, including lipid peroxidation, hyperlipidemia, free radical damage, and hyperglycemia, leading to qualitative and quantitative changes to the myocardium [1]. Treatment of ischemic injury includes restoration of the blood supply to ischemic tissue and prevention of damage inflicted at the time of injury.

Reactive oxygen species (ROS) play an important role in oxidative stress and related myocardial damage. Increasing numbers of ROS-like hydroxyl radicals and superoxide anions during heart ischemia lead to destruction of the cell membrane, development of lipid peroxides, and damage to the antioxidative defense system [2,3]. Experimental and clinical studies have shown that the infarct size of myocardial necrosis may be limited by the development of endogenous antioxidant enzymes and the suppression of free radical generation [4].

Post-MI myocardial damage is attenuated by anti-inflammatory, anti-fibrotic, and vasodilator agents. MI causes inflammation and leukocyte recruitment to the injured myocardium and renal glomerulus [5]. After MI, myofibroblast persistence may contribute to fibrosis and adverse myocardial remodeling, particularly if the myofibroblasts remain active in healthy areas of the heart that are located at a distance from the original site of injury (reactive fibrosis). Fibrosis in the remote myocardium inevitably leads to increased myocardial stiffness, resulting in systolic and diastolic dysfunction, left ventricular (LV) hypertrophy, arrhythmia, neurohormonal activation, and, ultimately, heart failure [6].

Recently, there has been growing interest toward establishing the therapeutic potential of natural products against several diseases. For instance, the consumption of plant items, such as antioxidant supplements or antioxidant-containing foods, might be used to protect against various diseases, including cardiovascular diseases [7]. *Euterpe oleracea* Mart., popularly known as “açaí,” is widely cultivated in the Amazon region of Brazil. Chemical studies have shown that this purple fruit contains hydroxybenzoic acids, antioxidant polyphenolics, flavan-3-ols, and anthocyanins, predominantly cyanidin 3-*O*-rutinoside and cyanidin 3-*O*-glucuronide [8-10]. Açaí exhibits anti-inflammatory action through the inhibition of cyclooxygenases 1 and 2 [11], vasodilator effect [12], inhibition of nitric oxide (NO) production, as well as inducible nitric oxide synthase (iNOS) activity and expression [13], and antioxidant properties in acute lung inflammation [14].

Although *E. oleracea* is an important medicinal plant that has antioxidant, anti-inflammatory, and vasodilator properties, its cardioprotective activity against MI has not been studied. Therefore, in this study, we investigated the effects of the oral administration of hydroalcoholic extract from the seeds of *E. oleracea* fruit on cardiac dysfunction and exercise intolerance of rats subjected to MI.

## Methods

### Preparation of extract from *Euterpe oleracea* (açaí)

*E. oleracea* Mart. fruit was obtained from Amazon Bay (Belém do Pará, Pará, Brazil) excicata number 29052 Museu Goeldi — Belem do Para. Hydroalcoholic extracts were obtained from the seed (stone), as previously described [14]. In brief, approximately 200 g of açaí stone were boiled in 400 ml of water for 5 min, mixed for 2 min, and then boiled again for 5 min. The decoction was cooled to room temperature and extracted by adding 400 ml of ethanol, with shaking for 2 h. The extract was stored in dark bottles at 4°C for 10 days.

After this maceration period, the açaí hydroalcoholic extracts were filtered through #1 Whatman filter paper. The ethanol was evaporated using a rotary evaporator (Fisatom Equipamentos Científicos Ltda São Paulo, São Paulo, Brazil) under low pressure at 55°C. The extract was lyophilized (LIOTOP model 202, Fisatom Equipamentos Científicos Ltda São Paulo, São Paulo, Brazil) at temperatures ranging from -30 to -40°C under vacuum at 200 mmHg. The extract was frozen at -20°C until use. Typically 100 g of stone yielded approximately 5 g of lyophilized extract. High-performance liquid chromatography (HPLC) analysis was performed as described previously [14].

### Animals

This study was performed on male Wistar rats (body weight: 150–200 g). Experimental protocols were approved by the Animal Care and Use Committee of the Federal University of Rio de Janeiro, Brazil.

### Coronary artery ligation

Under sevoflurane inhalation anesthesia (3%), a left thoracotomy was performed in the fourth or fifth intercostal space. While the heart was exteriorized, the anterior descendent coronary artery was ligated by 1–2 mm from its origin with a 6.0 suture [15]. In the sham group, the same procedure was employed, but the suture was not tied. Rats were divided into sham-operated and MI groups (6 rats per group), which either received treatment with *E. oleracea* seed hydroalcoholic extract (açaí, 100 mg/kg/day p.o. by gavage) or not. Treatment with açaí began on the day of surgery and lasted 4 weeks. The açaí hydroalcoholic extract was dissolved in water.

### Exercise test protocol

Four weeks after the surgery, rats performed a graded treadmill run to fatigue on a customized rodent treadmill (EP–131, Insight, São Paulo, Brazil). The protocol involved the rats running in three steps, with a progressive increase in treadmill speed: 1) 8 m min^-1^, 2) 12 m min^-1^, and 3) 18 m min^-1^. The two initial steps lasted for 3 min each, whereas rats continued running in the final step until they reached the point of fatigue, which was confirmed by the loss of the animal righting reflex. Subsequently, the total running distance was obtained.

### Cardiac mass and hemodynamic measurements

Four weeks after surgery, the animals were anesthetized with pentobarbital sodium (50 mg/kg, i.p.), and prepared for arterial blood and LV pressure measurements. A catheter (PE-50) was introduced into the right carotid artery, and arterial blood pressure was measured by PowerLab (ADInstruments, Sydney, Australia) monitoring equipment. Subsequently, the catheter was introduced into the LV to record LV pressure. Hemodynamic values were automatically calculated using a physiological data acquisition system LabChart 7.0 (ADInstruments, Sydney, Australia). Anesthetized animals were euthanized at the end of the experiment. Afterwards, their hearts were weighed, and the heart weight to body mass ratio was calculated.

### Histological analysis

The hearts of all rats from the experimental groups were harvested, instilled with 10% neutral buffered formalin, and immersed in the same fixative. Tissues were longitudinally cut, routinely processed, and embedded in paraffin blocks. Sections (7-μm-thick) were stained with picrosirius red and examined by optical microscopy. The volume fraction of collagen (%) was determined by measuring the area of stained LV within a given field. The stained area was calculated as a percentage of the total area within a field (Image-Pro Plus). Six fields were analyzed from each tissue and averaged.

### Statistical analysis

All data were expressed as the mean ± standard error of the mean (SEM). Differences among groups were considered statistically significant when the *P* value was <0.05 using one-way analysis of variance (ANOVA), followed by a post-hoc Dunnett’s test.

## Results

### Reduced exercise intolerance in MI rats with açaí treatment

**Figure 1:**
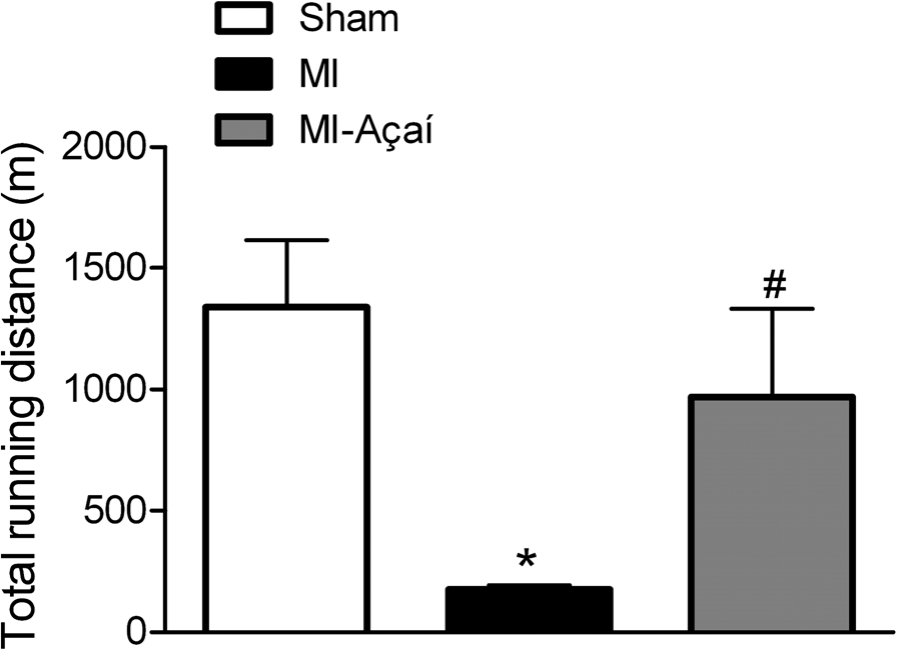
**Exercise test protocol.** Effects of the oral treatment with açaí extract (100 mg/kg) of MI rats during 4 weeks in exercise test protocol. Values are mean ± S.E.M. *P < 0.05 vs. Sham. # *P* < 0.05 vs. MI. n = 6 per group.

Four weeks after surgery, the animals were submitted to a treadmill test. The total running distance for sham rats was 1339.0 ± 276.6 m, compared to 177.6 ± 15.8 m for MI rats (*P* < 0.05 *vs.* sham) and 969.9 ± 362.2 m for MI-açaí rats (*P* < 0.05 *vs.* MI; Figure 1).

### Açaí administration normalizes MI rat hemodynamic and cardiac hypertrophy

**Figure 2:**
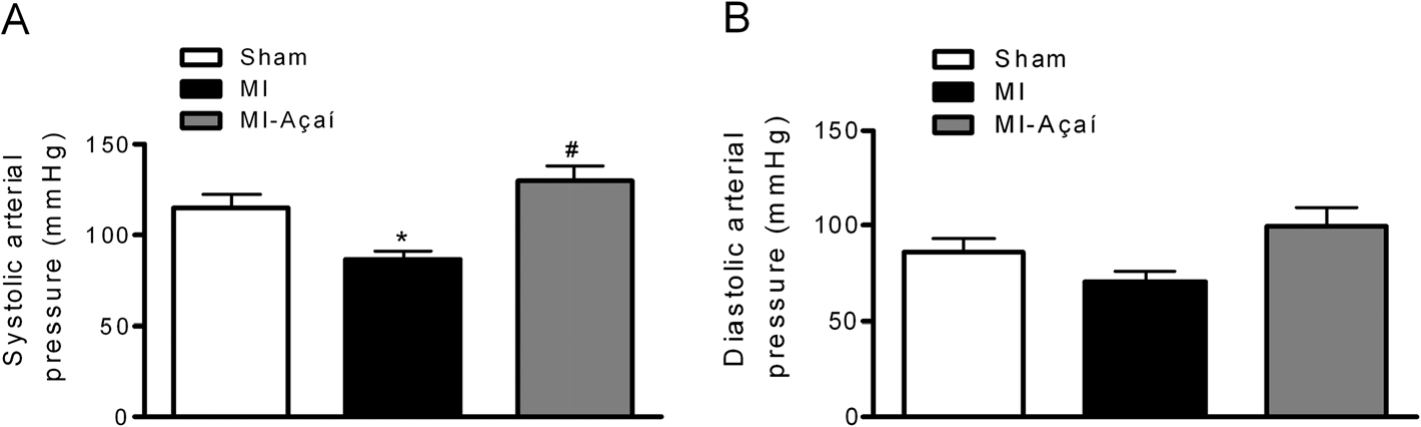
**Systolic and diastolic arterial pressures.** Effects of açaí extract (100 mg/kg) in systolic **(A)** and diastolic **(B)** arterial pressures of MI rats. Values are mean ± S.E.M. *P < 0.05 vs. Sham. # *P* < 0.05 vs. MI. n = 6 per group.

After oral treatment with açaí extract, rats were subject to hemodynamic analysis and cardiac hypertrophy evaluation. Systolic arterial pressure was decreased in MI rats (86.88 ± 4.62 mmHg) compared to sham rats (115.30 ± 7.24 mmHg; *P* < 0.05; Figure 2A). Açaí treatment prevented the reduction of systolic arterial pressure (130.00 ± 8.16 mmHg) compared to MI rats (*P* < 0.05 *vs.* MI; Figure 2A). The hemodynamic data showed no significant difference in the diastolic arterial pressure of the studied groups (Figure 2B).

**Figure 3:**
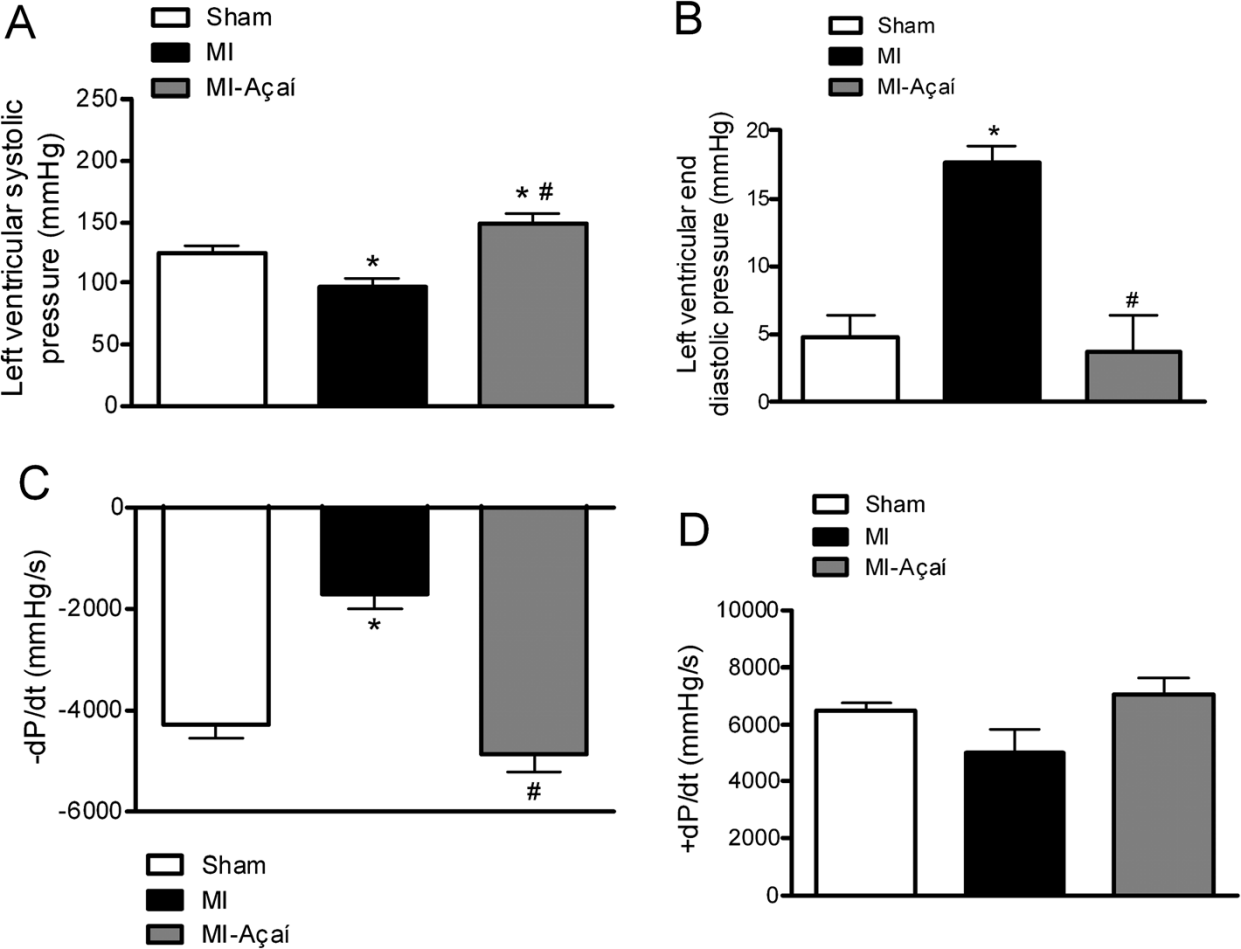
**Hemodynamic data.** Hemodynamic data of Sham, MI and MI rats treated with açaí extract (100 mg/kg) during 4 weeks. The analyzed parameters were **(A)** left ventricular systolic pressure, **(B)** left ventricular end diastolic pressure, **(C)** -dP/dt and **(D)** + dP/dt. Values are mean ± S.E.M. *P < 0.05 vs. Sham. # *P* < 0.05 vs. MI. n = 6 per group.

The LV systolic pressure of MI rats (97.29 ± 6.63 mmHg) was decreased compared to sham rats (124.20 ± 5.88 mmHg; *P* < 0.05) and açaí-treated rats (148.60 ± 8.47 mmHg; *P* < 0.05 *vs.* sham and MI; Figure 3A). The LV end-diastolic pressure was augmented in MI rats (17.62 ± 1.21 mmHg) compared to sham rats (4.15 ± 1.60 mmHg; *P* < 0.05), and it was 3.69 ± 2.69 mmHg in MI-açaí rats (*P* < 0.05 *vs.* MI; Figure 3B). The LV relaxation rate (assessed by -dp/dt) was reduced in MI rats compared to sham rats, whereas treatment with açaí prevented this reduction (Figure 3C). We did not observe any significant differences in LV contractility (assessed by + dp/dt) among the study groups (Figure 3D).

**Figure 4:**
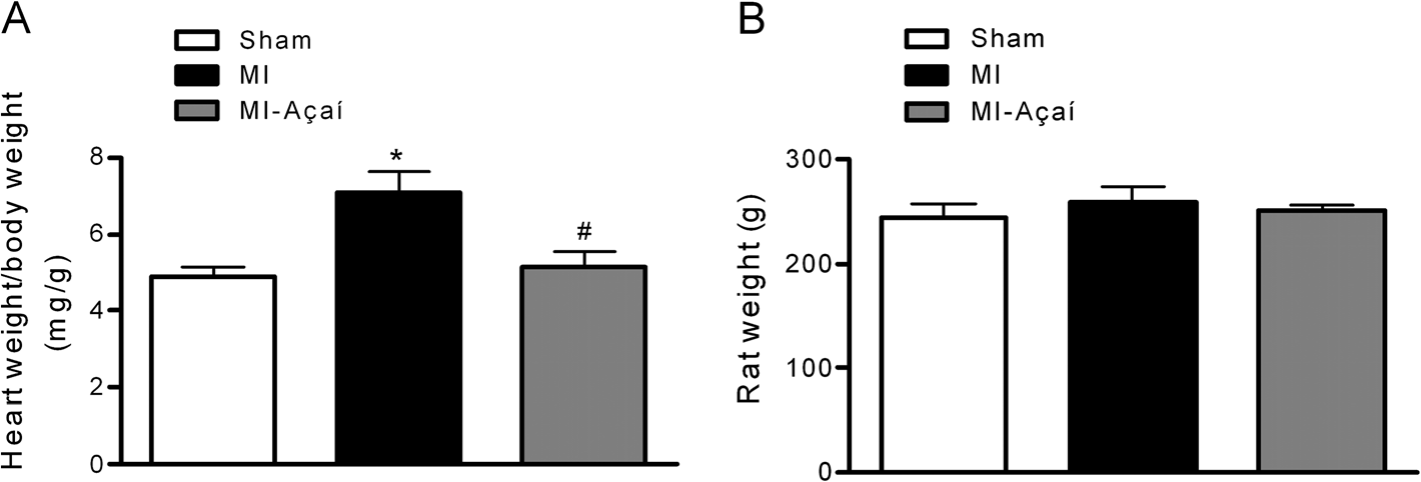
**Cardiac hypertrophy. (A)** Ratio of heart weight to body weight and **(B)** body weight of Sham, MI and MI rats treated with açaí extract (100 mg/kg) during 4 weeks. Values are mean ± S.E.M. *P < 0.05 vs. Sham. # *P* < 0.05 vs. MI. n = 6 per group.

The heart weight to body weight ratio was significantly greater in MI rats (7.07 ± 0.55 mg/g) compared to sham rats (4.89 ± 0.25 mg/g; *P* < 0.05). Açaí treatment prevented cardiac hypertrophy, with a heart weight to body weight ratio of 5.17 ± 0.37 mg/g (*P* < 0.05 *vs.* MI; Figure 4A). There was no significant difference in rat body weight among the experimental groups (Figure 4B).

### Reduction of LV fibrosis in MI rats after açaí extract treatment

**Figure 5:**
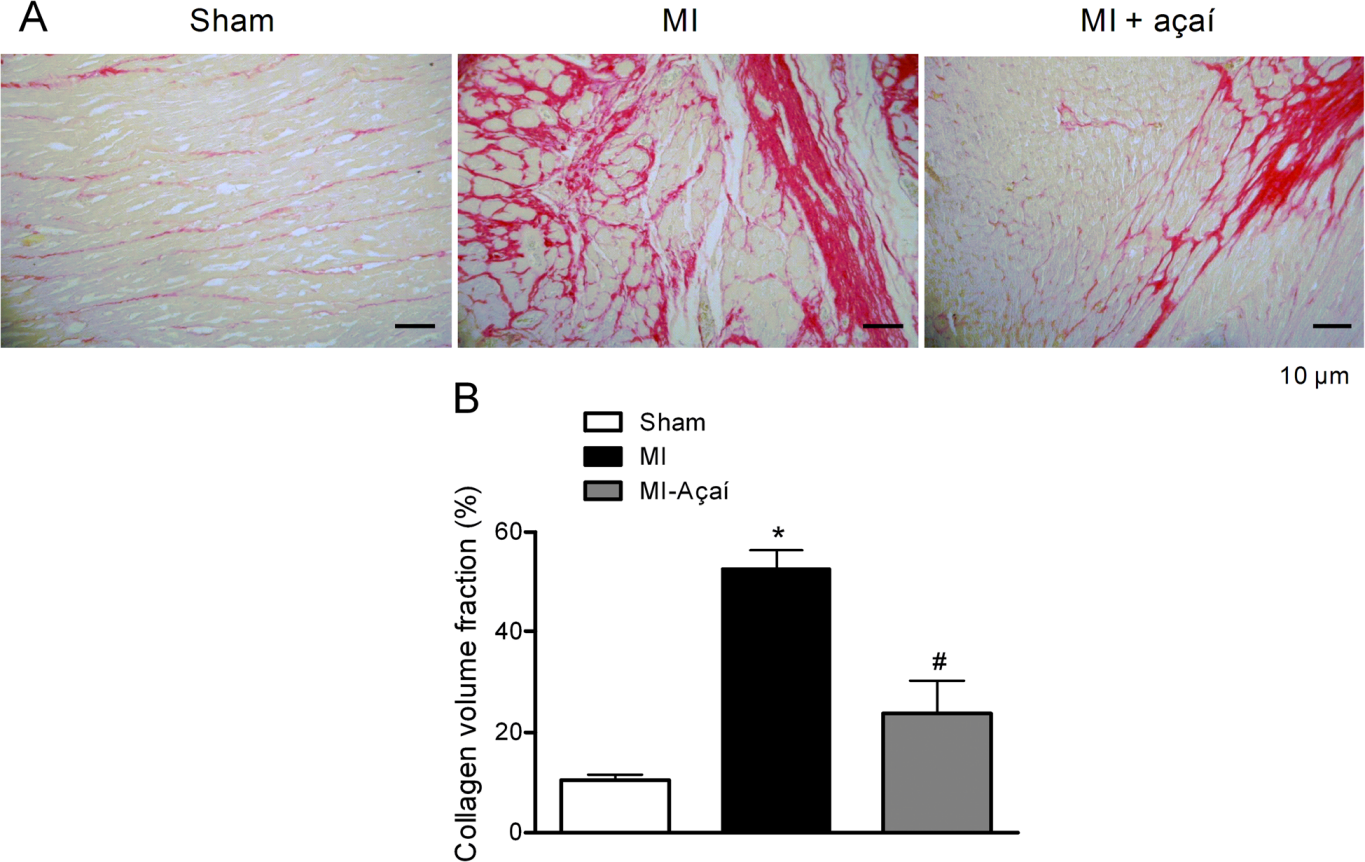
**Cardiac fibrosis.** Collagen volume analysis of the left ventricle from Sham, MI and MI rats treated with açaí extract (100 mg/kg) during 4 weeks. **(A)** Picrosirius red staining of left ventricle under light microscopy (magnification 20×), showing collagen fibers in red color. **(B)** Collagen volume fraction of left ventricle (%). Values are mean ± S.E.M. *P < 0.05 vs. Sham. # *P* < 0.05 vs. MI.

After the oral treatment of MI rats with açaí extract, collagen deposition in the LV was determined by picrosirius red staining (Figure 5A). The volume fraction of collagen (%) was 10.39 ± 1.06 in sham rats compared to 52.39 ± 3.85 in MI rats (*P* < 0.05). Oral treatment with açaí extract prevented LV fibrosis, with a collagen volume fraction (%) of 23.57 ± 6.78 (*P* < 0.05 *vs.* MI; Figure 5B).

## Discussion

In the present study, we demonstrated that the development of deleterious effects in MI rats was prevented by oral treatment with açaí extract for 4 weeks. Treatment with açaí prevented the reduction of exercise resistance during the exercise test procedure, in addition to preventing cardiac hypertrophy, LV fibrosis, and hemodynamic changes, such as reductions of systolic arterial pressure, LV systolic pressure, and relaxation rate (-dp/dt), and enhancement of the LV end-diastolic pressure.

In the exercise test protocol, MI rats ran for shorter distances compared to sham rats. Intolerance to physical exercise has been characterized in different animal models of heart failure [16], as well as in humans [17-19]. In this study, açaí extract treatment of the MI group for 4 weeks prevented a reduction in exercise tolerance, with rats performing at a similar level to sham rats. This beneficial effect might be partly related to the vasodilator action of açaí through the NO/sGC/cGMP pathway [12]. This action might improve blood flow in the skeletal muscle and, hence, exercise capacity. In addition, diastolic dysfunction is the primary mechanism responsible for dyspnea and muscle fatigue in heart failure subjects [20]. The role of açaí in preventing exercise intolerance might also be related to the delayed development of cardiac hypertrophy, reduction in the LV end-diastolic pressure, and an increase in the rate of relaxation (-dp/dt) of cardiac muscle from MI rats.

Plasma levels of proinflammatory cytokines seem to increase with heart failure, and have a predictive prognostic value [21]. MI induces leukocyte recruitment to the injured myocardium, which contributes to myocardial damage [5] which could be reduced by açai extract. Several studies have demonstrated the anti-inflammatory action of *E. oleracea*. More recently, Moura et al. [14] demonstrated that *E. oleracea* extract reduces acute lung inflammation in mice, by decreasing the numbers of alveolar macrophages and neutrophils in lung sections and decreasing TNF-α expression in lung homogenates. Another important action of *E. oleracea* in the controlling inflammatory process is the inhibition of NO production by reducing the expression of iNOS [13].

ROS and oxidative stress might also exacerbate myocardial damage after MI. Increasing numbers of hydroxyl radicals and superoxide anions during heart ischemia lead to destruction of the cell membrane, lipid peroxidation, and damage to the antioxidative defense system [2,3]. Experimental and clinical studies have shown that the infarct size of myocardial necrosis may be limited by antioxidant agents [4]. Several researchers have demonstrated the beneficial effects of açaí as an antioxidant agent [11,22-24]. Açaí juice increases the expression of antioxidant enzymes, such as glutathione reductase and glutathione peroxidase 3, in the aorta of apolipoprotein E-deficient mice.

Myofibroblast persistence after MI promotes fibrosis and myocardial remodeling, leading to increased myocardial stiffness, systolic and diastolic dysfunction, LV hypertrophy, arrhythmia, neurohormonal activation, and, ultimately, heart failure [6]. In the current study, the development of cardiac fibrosis was prevented in MI rats that were treated orally with açaí extract, based on the observed reduction of collagen deposition in the LV. Thus, treatment with açaí extract has beneficial effects in delaying cardiac remodeling, and represents a novel therapeutic agent to prevent heart failure resulting from MI.

## Conclusions

*E. oleracea* treatment for 4 weeks prevented the development of exercise intolerance, cardiac hypertrophy, fibrosis, and dysfunction in MI rats. These beneficial effects might be related to the antioxidant, vasodilator, and anti-inflammatory properties of its seed extract.

## Competing interests

The authors declare that they have no competing interests.

## Authors’ contributions

RTS, GZS and RSM idealized the study, helped to draft the manuscript, supervised the study design and revised the manuscript. JSS and SLP performed the pharmacological experimental work and analyzed the data. RSM, PJCS carried out the preparation of extract from *Euterpe oleracea*. All authors read and approved the final manuscript.

## Pre-publication history

The pre-publication history for this paper can be accessed here:

http://www.biomedcentral.com/1472-6882/14/227/prepub
